# Overview of mechanisms related to citrulline malate supplementation and different methods of high-intensity interval training on sports performance: A narrative review

**DOI:** 10.1016/j.heliyon.2025.e42649

**Published:** 2025-02-11

**Authors:** Hadi Nobari, Laya Samadian, Saber Saedmocheshi, Pablo Prieto-González, Christopher MacDonald

**Affiliations:** a LFE Research Group, Department of Health and Human Performance, Faculty of Physical Activity and Sport Science (INEF), Universidad Politécnica de Madrid, Madrid, Spain; b Department of Exercise Physiology, Faculty of Educational Sciences and Psychology, University of Mohaghegh Ardabili, Ardabil 56199-11367, Iran; c Department of Public Health, School of Public Health, Urmia University of Medical Sciences, Urmia, Iran; d Department of Physical Education and Sport Sciences, Faculty of Humanities and Social Sciences, University of Kurdistan, 66177-15175 Sanandaj, Kurdistan, Iran; e Sport Sciences and Diagnostics Research Group, Prince Sultan University, Riyadh 11586, Saudi Arabia; f Conway Medical Center College of Health and Human Performance, Coastal Carolina University, USA

**Keywords:** Amino acid, Ergogenic aid, HIIT, Nitric oxide, Physical fitness, Resistance training, VO_2_max

## Abstract

Regular exercise is a practical non-pharmacological approach to maintaining physical and mental health through rehabilitation and prevention of chronic diseases due to its cardiovascular, cardiorespiratory, neurological, and neuromuscular benefits. Despite awareness of the benefits of exercise, a “lack of time” has proven to be the most common impediment to regular activity. Thus, a time-efficient and potentially enjoyable training modality with growing popularity is high-intensity interval training (HIIT). HIIT incorporates intermittent bouts of work and recovery intervals performed at an intensity close to maximal oxygen consumption (VO_2_max). HIIT is considered to have equivalent or superior benefits compared to moderate-intensity continuous training (MICT). This narrative review focuses on the mechanisms of Citrulline Malate (CM) supplementation and various modes of HIIT on exercise performance. CM serves as a nitric-oxide enhancer leading to improved aerobic and anaerobic exercise performance by increasing muscle adenosine triphosphate (ATP) production, vasodilation, and blood flow to the active musculature and boosting work capacity. This article reviews the mechanisms related to CM supplementation and different modes of HIIT on exercise performance. Even though a single, acute 8 g dose of CM has been recommended, its mechanism of action remains to be seen due to the synergistic impact of both components (citrulline and malate). Moreover, the limited evidence for the standard level of supplement use and source of purchase results in athletes’ self-prescription of supplements. Therefore, to reduce the risk of accidental doping or toxicity, further studies should continue to investigate the optimal dose, timing, mechanism of action, as well as reliable sources of purchase for CM consumption.

## 1 Introduction

There is unquestionable evidence that regular exercise could help maintain physical and mental health throughout life [1], reduce the risk of morbidity [2], and mortality [3] primarily due to the prevention of chronic diseases. Current physical activity guidelines suggest 150 min of moderate or 75 min of intense physical activity per week [4]. Despite the well-documented benefits of exercise [5], one-third of adults and four-fifths of adolescents—about 1.4 billion people—do not meet the requirements of public health guidelines for recommended levels of physical activity [6]. Among several reasons reported for inactivity [7], a lack of time has proven to be the most common [8]. Consequently, powerful workout strategies with a lower time commitment are prioritized [9]. Interval exercise has been an evolving trend that might have benefits in this regard [3]. With the expansion of research in this field of study, it is suggested that interval exercise with higher intensities might offer more aerobic benefits than moderate-intensity continuous training (MICT) programs [10]. High-intensity interval training, known as HIIT, became popular among athletes in the 1980s and has been demonstrated to be one of the most effective training methods for improving athletic performance in various sports [11]. The evidence-based data indicate that HIIT is more than just a training tool; it can also be used to combat several inactivity-related disorders in different populations and improve elite athletes' exercise performance [12]. HIIT is defined as a series of repetitive intervals of short to moderate length (up to 5 min), with different work-to-rest ratios including 1:1, 1:0.75, 1:2, and 2:1 [13] done at an intensity between lactate threshold and maximal oxygen consumption (VO_2_max). Those repetitive intervals are punctuated by short and incomplete recovery periods. Therefore, HIIT relies on reducing training volume and increasing training intensity to stimulate improved performance [14]. VO_2_max is the highest level of oxygen consumption achieved in a specific graded exercise test [15] related to increased stroke volume (SV) [16], maximal cardiac output [17], capillary density, increased red blood cell volume, hemoglobin mass [18], and skeletal-muscle oxidative enzyme capacity [19].

Regarding the increasing popularity of high-performance sports and their relevant top-level competitions, small factors like discerning nutritional choices play a crucial role in the outcome of contests. Hence, anything that can provide a competitive advantage, including dietary supplements, can seem tempting [20]. Therefore, it is necessary to evaluate new evidence-based data about potent stimuli that reinforce the efficacy of HIIT for mixed athletic populations [11]. Targeted nutritional interventions can significantly affect performance during HIIT [11]. For example, it has been suggested to augment the relative amount of protein in athletes' diets to optimize anabolic processes and improve physiological responses to training and performance [21]. Accordingly, athletes often turn to supplement use to enhance their maximum performance [22]. With a usage frequency of 35–40 %, proteins and amino acids are the most commonly used supplements [23]. Amino acid supplementation leads to increased availability of essential amino acids, improved anabolic processes [24], promoting tissue growth, and expedited recovery rate during exercise in strength athletes. Recent studies have focused on nitric oxide (NO)-based signaling pathways [25]. The increase in NO levels caused by the consumption of ergogenic supplements leads to an increase in blood supply and mitochondrial function [26]. Many ergogenic supplements can increase NO levels, including beet juice and citrulline malate (CM) [25]. CM, an increasingly common ergogenic aid [27], is a combination of L-citrulline and malate and serves as an NO enhancer [28] leading to improved performance of high-intensity strength and power tasks [29] through increased vasodilation of blood vessels in the active musculature [30]. During exercise, increased blood flow to the working muscles and mitochondrial activity can prolong exercise and delay fatigue. Increasing nitric oxide production, either endogenously or through dietary supplementation, can improve performance. There are many sources that increase NO synthesis, including beets, citrulline, and the compound citrulline malate [26]. Another similar mechanism, which describes the effect of citrulline malate consumption on nitric oxide production, is related to the signaling pathway via the L-arginine NO pathway, which, with nitric oxide production from supplement consumption, leads to the relaxation of capillary and vascular smooth muscle, resulting in vasodilation [31]. Also, although NO production may be reduced in the conversion of citrulline to arginine, arginine production itself can act as a vasodilator, leading to NO production resulting in increased blood flow. The presence of two important substances, citrulline and malate in the CM supplement can have a possible synergistic role at the muscle level [32]. In the chain of energy production, malate can play a role in energy generation and help to continue the path of energy generation [32]. In addition, malate can contribute to the aspartate malate shuttle [33]Although a single acute 8 g dose of CM has been the most common approach so far, equivocal research results have been reported [28], leaving it unclear whether the effects of CM are due to L-citrulline or malate or a synergistic effect of their combination, challenging the effectiveness of CM on exercise performance [34].

Therefore, further studies on optimal dosage are required to quantify the bioavailability of NO, citrulline, and malate after taking a series of amounts of CM. Contradictory cross-sectional studies have reported citrulline malate supplementation and mentioned its possible mechanism of action to increase blood supply and increase excretion of [30,35]. Fast recovery and disposal of waste metabolites can prepare the athlete for the next competition. This possible mechanism could be beneficial for athletes. However, since most of the studies conducted on human samples have been on inactive and sedentary people, and there is little information about the effect of taking this supplement in the short and long term in athletes, the current study examines the possible mechanisms of citrulline malate during exercise and its possible ergogenic role. Recently, there has been increasing publicity about citrulline malate, which can lead to improved performance through the production of nitric oxide. This review article attempts to examine the effectiveness of taking this supplement on athletic performance, both from a molecular and functional perspective. It has also been noted that citrulline malate supplementation, due to the presence of malate and its involvement in the energy production pathway, can increase the rate of ATP production by reducing lactate production. This action occurs through the presence of a shuttle in the electron transport chain and the continuous production of pyruvate [36]. Likewise, further well-controlled studies with highly repeatable training protocols with a significant aerobic component are needed to evaluate the mechanisms associated with this supplement properly. Until such studies are completed, the efficacy of CM supplementation for enhancing exercise performance remains equivocal [28]. Moreover, there is little information on how athletes approach supplement use and purchase. Even though proteins and amino acids are the most consumed ergogenic supplements, there is little scientific evidence for standardized intake, leading to athletes prescribing themselves without consulting a credentialed professional. Additionally, not all athletes know the contamination risks [37] that dietary supplements can pose and may be exposed to accidental doping [38]. Therefore, there is an urgent need to provide nutritional education and advice emphasizing the roles of nutritionists and sports scientists along with the genuine and long-term side effects of faulty nutrition regimens [23].

## 2 Methodology

### 2.1 Study design

This manuscript is a narrative review that aims to provide an overview of the mechanisms related to CM supplementation and different methods of HIIT on sports performance. This review synthesizes existing literature and presents an analysis of the current evidence regarding the effects of CM supplementation and various modes of HIIT on exercise performance.

### 2.2 Research strategy

This narrative review was conducted to provide an overview of mechanisms associated with CM supplementation and different methods of HIIT on exercise performance. The research strategy involved searching electronic databases, including PubMed, Scopus, and Google Scholar, using appropriate search terms related to CM supplementation, HIIT, exercise performance, and ergogenic aids. The keywords included: amino acid; ergogenic aid; HIIT; nitric oxide; physical fitness; resistance training; VO_2_max. The search was limited to articles published in English. Additionally, the reference lists of the retrieved articles were reviewed to identify additional relevant studies.

### 2.3 Inclusion and exclusion criteria

Studies were included in the review if they met the following criteria: (1) investigation of the effects of CM supplementation on exercise performance, (2) examination of the effects of different modes of HIIT on exercise performance, (3) human participants, and (4) reporting of relevant outcomes such as aerobic capacity, anaerobic performance, or other measures of exercise performance. Studies focusing on other forms of exercise or interventions not directly related to CM supplementation or HIIT were excluded. There were no restrictions on the publication date or availability of the full text.

The extracted data included study design, sample size, participant characteristics (e.g., age, fitness level), intervention details (e.g., CM dosage, HIIT protocol), and outcomes related to exercise performance.

### 2.4 Limitations

This narrative review has certain limitations. First, the inclusion of studies was limited to those published in English, which may introduce language bias. Second, the review focused on published literature, and unpublished studies or ongoing research were not considered. Finally, the conclusions drawn from the review are based on the synthesized evidence and individual study findings may vary.

## 3 High-intensity interval training

Interval training is generally defined as recurring bouts of moderate to high-intensity exercise separated by periods of passive or active recovery [39]. It has been implemented by coaches and athletes for over a century, resulting in improved exercise performance [40].

The fitness industry as a whole has recently experienced increased interest in HIIT, a cycle of burst and recovery [41] often performed with an “all-out” attempt at an intensity close to VO_2_max (i.e., ≥90 % of VO_2_max) [15] or >75 % of maximal power, ≥90 % minimal running speed required to elicit VO_2_max, ranging from “hard” to “very hard” rating of perceived exertion (≥6 on 10 point scales and ≥15 on 6–20 point scales) [6]. Similarly, MICT can enhance cardiovascular fitness, reverse metabolic syndrome risk factors, reduce body fat, reduce total cholesterol [42], and has been proposed as an alternative to the traditional approach for improving aerobic fitness. Although both MICT and HIIT lead to noticeable improvements in VO_2_max as opposed to no exercise [41], some studies indicate that HIIT leads to greater improvements in both aerobic and anaerobic fitness [43] compared to endurance training alone [43]. Despite not knowing the exact mechanisms leading to these substantial HIIT adaptations, it is suggested that they might be related to exercise time spent at or near VO_2_max, higher levels of muscle fiber recruitment, related cardiovascular system activation [4] or the different molecular pathways activated by the applied interval training protocols [6]. For example, an endurance training focus that includes HIIT can result in the physiological stimuli for mitochondrial biogenesis, leading to reduced glycogen consumption, reduced lactate production, and increased lactate threshold resulting in more extended periods of exercise at a particular intensity. On the other hand, a strength training focus that includes HIIT will more readily stimulate synthesis of myofibrillar proteins resulting in muscle hypertrophy and increased peak strength [6].

### 3.1 Physiological benefits of HIIT

Given the significant benefits of HIIT for enhancing cardiorespiratory fitness [44], such as improved VO_2_max and improved skeletal muscle oxidative capacity, research in clinical populations has indicated that HIIT programs with higher volumes and longer intervals provide more significant improvements in VO_2_max than MICT at similar volumes and durations [4,45]. De Revere et al. [46] reported that three weeks of nine HIIT sessions yielded enough stimuli to increase cardiac output and VO_2_max [6]. Similarly, Daussin et al. [47] found that the increase in VO_2_max was greater for untrained men and women participating in an eight week HIIT program (15 %) than for untrained participants taking part in an endurance training program (9 %) [41]. The vascular health and function-related benefits of aerobic exercise are associated with endothelial cells [48] in the vasculature releasing NO, which has a potent vasodilatory effect that protects cardiovascular function and health. Studies have reported the beneficial effect of HIIT on the proper functioning of these endothelial cells, leading to efficient blood flow distribution and improving arterial vascular elasticity. These results appear consistent in various cardiovascular disease conditions [4,48]. In the mid-1990s, Meyer et al. [[49], [50], [51]] employed a variety of HIIT protocols on patients with heart failure and, for the first time, suggested an interval training model could be more suitable for improving heart function and physical performance in patients with chronic congestive heart failure [6]. Likewise, in 2004, Rognmo et al. [52] investigated whether 10 weeks of HIIT in patients with coronary heart disease led to similar or greater increases in VO_2_max compared to MICT. Their results indicated an average increase of 17.9 % in the HIIT group vs. 7.9 % in the MICT group.

Furthermore, muscle glycogen replenishment improves metabolic health via better insulin sensitivity and more optimal glucose control [4]. Unlike regular exercise, it is well established that inactivity reduces tissue sensitivity to insulin, causing impaired glycemic control, pancreatic cell damage, and development of type-2 diabetes and related conditions (e.g., metabolic syndrome, and prediabetes). Therefore, studies have suggested HIIT as an alternative to MICT for improving metabolic health and insulin sensitivity [6,53]. Although as little as two weeks of HIIT have been shown to improve aerobic fitness in healthy adolescents [54,55], evidence regarding the metabolic health benefits of HIIT in adolescents is currently bound by more extended periods of exercise (7–12 weeks) [55]. Additionally, less rigorous HIIT protocols could be more convenient for people with metabolic diseases and ultimately improve insulin sensitivity and blood sugar control [4,56].

### 3.2 Psychological benefits of HIIT

Even though many studies have assessed the physical health benefits of aerobic HIIT, there is little information on how it is perceived, whether participants enjoy it or not, and most significantly whether participants would comply with HIIT over time. Knowing how individuals perceptually and behaviorally respond to HIIT is crucial to encourage clients and patients to use this exercise approach. The following sections examine what is currently known about the effects of aerobic HIIT training on emotion, pleasure, and compliance [4].

#### 3.2.1 Affect

“Affects” are reactions perceived reflexively or instinctively without much thought and are associated with pleasure, discomfort, tension or tranquility [4]. Changes in mood and sentiment can fluctuate over time during workouts, meaning one can feel good and bad several times during the workout [2]. In particular, when clients or patients experience any aversion to training (e.g., pain, discomfort, stress), even HIIT might not be a viable training approach [4].

#### 3.2.2 Enjoyment

While an instinctive mood response is attributed to “affect”, “enjoyment” is a specific psychological condition drawn by a situational or cognitive appraisal. “Enjoyment” considers the answers to questions such as: Do I feel accomplished? Do I feel revived or refreshed? Or, more generally, did I enjoy my exercise experience? Recent data suggests that HIIT elicits higher enjoyment than continuous moderate-intensity and vigorous-intensity exercise [4,57].

#### 3.2.3 Adherence

In addition to the previously mentioned responses to HIIT, long-term exercise adherence is equivocal. A limited amount of data indicates that exercise adherence is better predicted through affective responses during exercise than at the end of the training [4].

Jung et al. [2] provides preliminary results in this regard, reporting that even though HIIT leads to less displeasure than continuous vigorous-intensity exercise (CVI) and less pleasure than continuous moderate-intensity exercise (CMI) in a sample of inactive adults, it is perceived as more enjoyable and is preferred over CVI and CMI.

HIIT can be implemented during aerobic, anaerobic, and resistance training modes with various compliant variables such as exercise intensity and duration, recovery intensity and duration, exercise modality, repetitions, and the number of series [10]. However, determining an optimal exercise regimen to improve fitness requires knowing how adaptations within physiological parameters are affected [58] by HIIT.

### 3.3 HIIT training modalities

Given the initial description regarding HIIT, remarkable attention has been drawn to the best high-intensity protocol to produce significant results with less time commitment and low volumes of exercise [10]. ‘‘Aerobic HIIT’’ and ‘‘body weight/resistance HIIT’’ are two general categories that have emerged. Both variations include bouts of intense exercise followed by recovery periods, with the main difference being the modality. Regarding aerobic HIIT training, the most common modes are running and cycling, where the desired intensity is reached through activities such as spin classes or track-based running workouts. In contrast, resistance/body weight HIIT uses calisthenics, plyometric, and/or weighted objects in programs such as Tabata, CrossFit, or Bootcamp training. Even though both types of HIIT programs are widely used, most research has targeted aerobic HIIT since cycling and treadmill running allow for a more precise performance evaluation to account for the training stimulus [4]. The growing body of research on aerobic HIIT should give practitioners more assurance of optimal prescription [4]. The following describes a few aerobic HIIT and body weight/resistance HIIT methods that differ in intensities, durations, and total exercise volume.

#### 3.3.1 Wingate model

Much of the current focus of HIIT is tied to an approach involving multiple trials of the Wingate anaerobic test, which measures peak anaerobic power and anaerobic capacity [59]. The Wingate-based HIIT includes 30 s of maximal volitional intensity, followed by approximately 4 min of recovery, repeated 4–6 times. For instance, using Wingate-based protocols in HIIT studies requires the athlete to perform 30 s of complete supramaximal cycling separated by 4 min of no-load pedaling. Since this HIIT training model takes less total work and less time to train, it is usually called “low-volume HIIT”. Notably, this approach to HIIT was primarily developed to indicate the effectiveness of interval training in producing rapid cardiometabolic adaptations and is generally not the recommended exercise style for a long-term program [4].

#### 3.3.2 Scandinavian model

This Scandinavian HIIT model was developed for compatibility with cardiac patients and included multiple 4 min intervals with similar periods of quick recovery [45]. Therefore, this training style is called “high volume HIIT” since the total time spent in vigorous exercise is usually more than 15 min, making it comparable to traditional endurance training modes. It is essential that the intervals are performed slightly below the maximum heart rate and consequently do not constitute a maximal volitional workout. The results of relevant studies have revealed substantial cardiovascular advantages of HIIT compared to standard aerobic exercise [4].

#### 3.3.3 Practical model

A new time efficient modification of HIIT, which developed recently as a substitute for the all-out intensities associated with the Wingate model [4,60], includes intervals on a stationary exercise bike for 60 s at the near-maximal intensity and alternates with light-intensity rest intervals of equal duration. Since this approach to interval training is somewhat of a midpoint between the two previously mentioned models (in terms of intensity, recovery, and total volume), it is a practical model that can be regarded as a “moderate volume” interval training program [4].

#### 3.3.4 Tabata training

Tabata is a type of HIIT workout named after its founder Izumi Tabata and aims to provide the most benefits in as little time as possible. This training consists of eight sets of 20-s work intervals performed at 170 % of the subject's VO_2_max, with 10-s recovery intervals and a total duration of 4 min [10].

### 3.4 HIIT program optimization

Despite the limited knowledge available on optimizing HIIT programs, optimization in the current context refers to the type of recovery (active vs. passive) and duration between training units, the optimal training intensity, training durations, and the number of interval units [30]. Studies have indicated several variables used to prescribe exercise intensities for individuals, including VO_2_max, anaerobic threshold, lactate threshold, ventilation threshold, the onset of blood lactate accumulation (OBLA), and critical power. Consideration of the appropriate and efficient exercise intensities should be determined by the physiological importance, feasibility, and rationale of using such measures [4,43].

### 3.5 HIIT safety considerations

Despite the health benefits of HIIT, engaging in this exercise requires practitioners to consider established guidelines regarding risks. In particular, this exercise modality is recommended for low-risk individuals, physician-approved moderate-risk individuals, and high-risk individuals under direct medical supervision during training [4]. Additionally, coaches and athletes should be knowledgeable about HIIT program optimization. Currently, the intensity, duration, and recovery of a HIIT program that produces the ideal rate of improvement [43] are yet to be precisely understood.

## 4 Supplementation and HIIT

This section focuses on how best to use HIIT to prepare trained athletes for competition [43]. When considering factors such as talent, training, tactics, and motivation [20], many athletes of all performance levels attach great importance to dietary supplements [20]. The physiological vasoactive gaseous signaling molecule, NO, is one supplement that has recently been the subject of much research [25]. Increased NO synthesis by exogenous substances can improve skeletal muscle function and performance through increased blood vessel vasodilation, contractility, mitochondrial respiration, glucose uptake, and calcium handling in the sarcoplasmic reticulum [28,29].

A popular class of dietary supplements includes NO precursors. Given the effects of NO on a wide range of exercise-related physiological processes, nutritional supplements containing NO precursors are often marketed to athletes and other active populations engaged in high-intensity exercise [29]. Supplementation with arginine and its precursor, citrulline (at various doses), as substrates for NO production has been examined [61] in the treatment of endothelial dysfunction, arterial hypertension, pulmonary arterial hypertension, pressure sores, erectile dysfunction, arteriosclerosis, some mitochondrial disorders, and necrotizing enterocolitis [62]. Citrulline is an increasingly popular dietary supplement with antioxidant and vasodilation properties and it is believed to improve athletic performance by augmenting NO production [63]. Citrulline's metabolic properties were effectively neglected until the last decade when it emerged as a supplement with various regulatory properties and it plays an essential role in nitrogen homeostasis [64].

Researchers have also examined the effects of L-arginine supplementation on exercise outcomes [29]. A study by Liu et al. [65] investigated the effect of 6 g of arginine per day for three days on recurrent cycling performance in trained judo athletes, finding no ergogenic effect [29]. Another study by Sunderland et al. [66] also observed no effect of 4-week L-arginine supplementation on VO_2_max and respiratory threshold in trained cyclists [29]. Specifically, since the bioavailability of oral L-arginine supplementation is estimated to be approximately 60 % [29], studies in trained athletes observed that oral L-arginine led to no significant increase in markers of systemic NO production [65,67,68]. On the other hand, oral supplementation with L-citrulline as a natural L-arginine precursor is better tolerated than arginine and may help improve muscle strength or protein balance [61]. The better bioavailability of L-citrulline, compared to L-arginine, is primarily due to its avoidance of first-pass hepatic metabolism and a longer circulation time [62].

Further comparisons in the literature [69] indicate gastrointestinal side effects of oral L-arginine supplementation, including nausea, abdominal cramps, and diarrhea. No side effects were observed with oral administration of citrulline [70]. No toxicity was noted when infants and children were given oral citrulline at doses up to 3.8 g/m^2^ daily (in five doses of 1.9 g/m^2^ every 12 h) [61] or in amounts up to 15 g in healthy human adults [71]. Due to limited breakdown in the placenta, it seems to be a recommended supplement for pregnant women [61]. A study by Powers et al. [72] in 24 healthy obese pregnant women supplemented with citrulline at a dose of 3 g/day for 3 weeks starting from 16 weeks gestation reported improved vascular function and lower blood pressure (108.1 ± 4.2/65.8 ± 6.6 mmHg) compared to placebo-treated obese women (116.1 ± 6.4/72.2 ± 6.2 mmHg, *p* < 0.05) [61].

### 4.1 Citrulline malate

A common form of citrulline supplementation is CM. This organic salt is formed through the combination of L-citrulline (C6H13N3O3), a non-essential amino acid involved in the urea cycle and malate (or malic acid, C4H6O5), an intermediate in the tricarboxylic acid (TCA) [28] which are combined in ratios ranging from 1:1 to 2:1 [29].

#### 4.1.1 Effects and ergogenic mechanisms

**Fig. 1:**
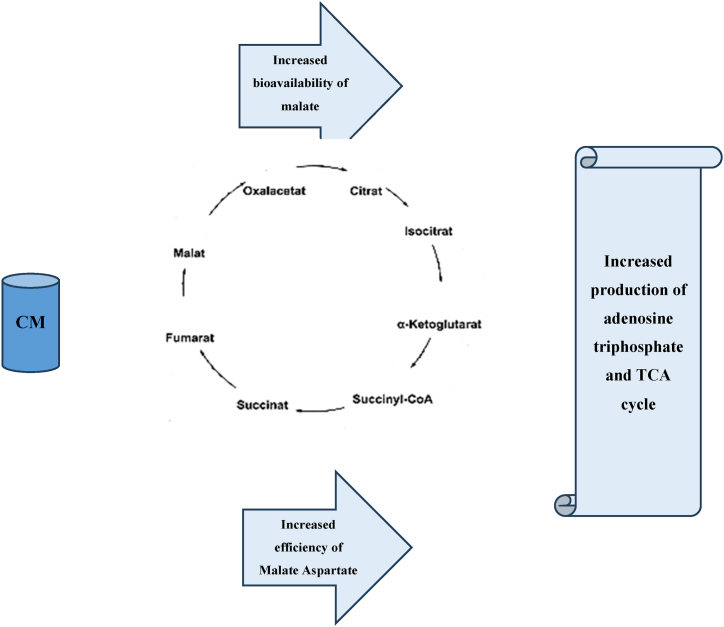
Two possible mechanisms in the effectiveness of citrulline malate supplementation on sports performance.

The effects of citrulline may be attributed to the effects of NO on blood flow, energy efficiency, contractility, muscle function, mitochondrial respiration [63,73], ammonia clearance, and aerobic adenosine triphosphate (ATP) production [74]. The proposed mechanism for CM uptake relies initially on the citrulline component through the L-arginine NO pathway, allowing the smooth muscle to relax after NO synthesis, resulting in vasodilation. These vasodilatory properties can improve the oxygen and blood flow to and from active muscles during exercise [28]. Trexler et al. [29,75] have recently challenged this mechanism to precisely detect changes associated with supplement consumption by using the reliable near-infrared spectroscopy (NIRS) technique [28] which has not yet proven to be sensitive enough in tracking the relevant changes, though. Two possible mechanisms in the effectiveness of citrulline malate supplementation on sports performance can be seen in the image below (see Fig. 1).

**Fig. 2:**
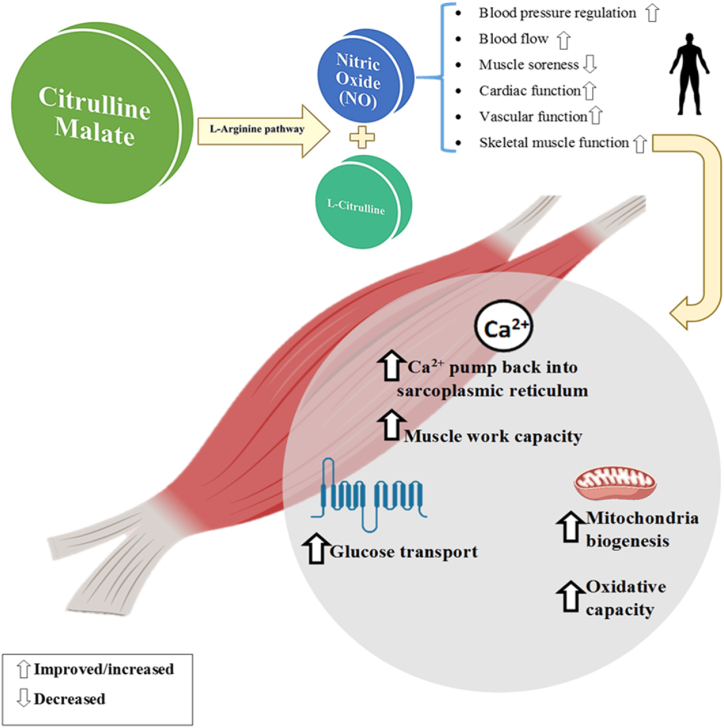
Overview of the mechanisms associated with CM supplementation and its effects on exercise performance (Created with BioRender.com).

Higher rates of ATP production during CM supplementation have been demonstrated in active human skeletal muscle using magnetic resonance spectroscopy [76]. Malic acid has been suggested to increase the rate of ATP production by attenuating lactate production under high-flow conditions. This allows for continuous pyruvate and energy production. In addition, the malate-aspartate shuttle (MAS) may become more efficient after CM ingestion, increasing ATP availability [77]. According to these promising results and additional mechanisms compared to L-citrulline supplementation alone, it is conceivable to suggest that CM supplementation may be a valuable performance-enhancing ergogenic aid [28]. An early study from 2002 by Bendahan et al. showed that chronic CM intake for 15 days (6 g/day) increased the contribution of oxidative ATP synthesis (34 % increase) to energy production [78]. A study in men who self-reported fatigue demonstrated a significant increase in aerobic ATP production and phosphocreatine recovery during finger flexion exercise [78]. However, this study focused on sedentary individuals who complained of fatigue and did not include a placebo condition, limiting its applicability to athletes [28]. On the other hand, studies on trained cyclists using 6 g of CM supplementation showed increased NO metabolite production after exercise [79]. In 2010, Perez-Guisado and Jakeman [74] conducted the first resistance training study with CM. A single 8 g dose of CM taken 1 h before resistance training had significantly increased the number of bench press repetitions performed over a 16-set workout and led to a 39.7 % decrease in muscle soreness after 24 h and a 41.8 % decrease in muscle soreness after 48 h compared to the placebo condition. Similarly, a reduction in muscle soreness was reported in a study by Chappell et al. [80] 24, 48, and 72 h post training. Conversely, a study by da Silva et al. [81] in nine recreationally active men consuming a 6 g dose of CM indicated no significant change in muscle soreness. Given the ambiguous responses to CM supplementation that have been reported, rendering the potential for performance enhancement unclear. Trexler et al. reported that 8 g intake of CM 2 h before exercise did not affect muscle blood flow (CM: 3.78 ± 0.26 ml min^−1^.100 ml^−1^; placebo: 3.72 ± 0.26 ml min^−1^.100 ml^−1^) or oxygen consumption (CM:1.15 ± 0.11 mlO_2_.min^−1^.100 g^−1^; placebo: 1.16 ± 0.11 mlO_2_.min^−1^.100 g^−1^) during a leg extension exercise for 3 min (at a rate of one repetition every 4 s) [28]. Similar findings were again reported by Trexler et al. [82] during five sets of 30 maximal effort concentric leg extension exercise with 1 min of passive rest between sets. These results were at least in part due to the nominal increase in NO values observed in these studies, as it was not remarkably different from the placebo (CM: 15.3 ± 1.1 μmol L; PLA: 13.4 ± 1.1 μmol L) [28,75,82]. Alternatively, if dynamic whole-body exercise was not chosen, the aerobic mechanisms associated with taking this supplement might not have been sufficiently stressed, resulting in no improvements being observed [28]. Another mechanism may involve the citrulline component of CM as this may help remove ammonia during the urea cycle [78]. An overview of the mechanisms associated with CM supplementation and its effects on exercise performance is illustrated in Fig. 2.

Citrulline malate is used to help eliminate ammonia in the urea cycle [83]. High-intensity, high-duration exercise leads to fatigue, which can lead to activation of the ammonia toluene cycle and lactate production, and reduced ATP production. Citrulline malate activates the energy transport chain and cycle [84]. High-intensity exercise increases ammonia levels in skeletal muscle. Ammonia activates phosphofructokinase and prevents pyruvate from oxidation to acetyl-CoA, leading to fatigue [85]. High concentrations of ammonia activate PFK, promoting lactic acid production during anaerobic glycolysis. This interferes with the oxidative metabolism of pyruvate to acetyl-CoA and prevents the supply of ATP to skeletal muscle. Citrulline may have its ergogenic effects through this mechanism by detoxifying ammonia during high-intensity exercise, thus leading to improvements in aerobic utilization of pyruvate and skeletal muscle ATP delivery [28,84]. Takeda et al. [85] reported that citrulline intake (250 mg kg^−1^ BM) increased ammonia accumulation to 90.1 μg/dl^−1^ (citrulline: 351.3 ± 35.3 μg dL^−1^ vs. control: 441.4 ± 61.3 μg dL^−1^) and prolonged swimming time to exhaustion by approximately 9 min in mice (citrulline: 24 min vs. control: 15 min). Importantly, post-exercise lactate levels were lower in the citrulline-supplemented group, supporting this supplement and its mechanisms improve aerobic exercise utilization. In addition, taking CM supplements through increasing blood flow and vasodilation leads to a reduction in muscle pain and lactate accumulation and delayed bruising [74]. Notably, it has not yet been confirmed whether this mechanism occurs during human exercise [28]. After all, the mechanisms of action of CM might not be fully known due to the synergistic impact of both components (L-citrulline and malate) at the intramuscular level [28,86]. Consequently, further exploration is needed to understand a complete overview of the [64] CM mechanisms.

#### 4.1.2 Dose, timing, and safety

The most commonly used dose of CM is a single acute dose of 8 g [87], which seems to reflect early work in which performance benefits were observed during resistance training at this dose [88]. However, a previous dose-response study [71] examined the pharmacokinetics of 2, 5, 10, and 15 g of citrulline in eight healthy volunteers and showed that larger doses might be more compatible. According to the authors’ report, the maximum concentration of citrulline occurred at a dose of 15 g (3849 ± 190 μmol l^−1^), which was significantly higher (+28.4 %) compared to 10 g of citrulline (2756 ± 170 μmol l^−1^). It is reasonable that such higher doses (>10 g) increase the likelihood of obtaining ergogenic benefits during exercise, calling into question the use of 8 g doses in most previous research [28]. There seem to be limited variations for the timing of CM consumption, with most studies opting for consumption 1 h before exercise [84]. Nevertheless, in a survey by Moinard et al. [71], peak citrulline concentration occurred at approximately 1 h after a series of doses of citrulline (2, 5, 10, and 15 g), but declined 15–30 min after the first peak regardless of the amount taken. Further research examining temporal changes in citrulline concentration after CM supplementation is needed to determine the optimal strategy, although the best recommendation is currently 1 h before exercise [28]. Even though most studies examine CM intake and exercise performance, the 2:1 ratio of citrulline to malate has been primarily implemented. Recent studies have questioned these reports, with many CM manufacturers failing to achieve the claimed ratios [89]. Significantly, a magnetic resonance spectroscopy study reported a CM ratio of about 6:1, with some as low as 1:1 provided by nutritional companies/suppliers leading to 4.2 g ingestion of CM instead of 5.3 g (the latter based on a 2:1 ratio) [28]. In a study by Brendahan et al. [78], a group of 18 sedentary men was chronically supplemented with 6 g.day^−1^ CM for 15 days and reported no adverse experiences. Nonetheless, no objective health markers were measured, and the reports were subjective opinions of the authors. On the other hand, Casonatto et al. [90] reported that after 6 g CM supplementation, both diastolic and systolic blood pressure were reduced 24 h after exercise (40 min of running/walking at 60–70 % HRR^1^ in a group of 40 hypertensive participants. Application of these discoveries would not readily apply to athletes as they are likely to undertake a more systematic use of CM compared to hypertensive individuals, and thus, further research has to be done regarding the safety of longer-term addition of CM [28]. Table 1 summarizes the studies evaluating the effects of citrulline malate on resistance exercise performance with different doses and timing.

**Table 1:** Characteristics of studies analyzing the effects of citrulline malate on resistance exercise performance.

Authors	Sample	Dose and Timing	Protocol	Results
Perez-Guisado et al. [74]	-REC males (n = 41) -Age: 30 ± 8 years	- CM: 8 g, single dose, 1^−h^ prior to exercise - PLA: 10 g sugar and 60 mg sodium saccharine, single dose, 1^−h^ prior to exercise	- RDB, crossover design (7-day washout) - used to test dynamic concentric and eccentric muscular endurance/ Strength using barbell. during 4 sets at 80 % 1RM until failure before and then again after a pectoral training workout with 1-min rest between sets of barbell bench press RE	• Significant increase in the number of bench press repetitions performed over a 16-set workout. • 39.7 % decrease in muscle soreness following 24-h compared to PLA • 41.8 % decrease in muscle soreness following 48-h vs. PLA
da Silva et al. [81]	- REC males (n = 9) - Age: 24 ± 3 years	- CM: 6 g, Single dose, 1-h prior to exercise - PLA: Corn starch, single dose, 1-h prior to exercise	- RDB crossover design (7-day washout) - used to test recovery of dynamic muscular endurance during 1 set at 100 % of 10 RM, Machine Hack Squat and Machine Leg press of RE	• No significant change in lactate, creatine kinase and muscle soreness
Chappell et al. [80]	- Recreational RT males (n = 12) and females (n = 17) - Age: 26 ± 8 years	- CM: 8 g, single dose, 1-h prior to exercise - PLA: 6 g Citric acid, single dose, 1-h prior to exercise	- RDB, counter balanced, crossover design (7-day washout design - placebo - controlled, used to test dynamic concentric and eccentric muscular endurance/strength using barbell during 10 sets × 10 reps at 80 % 1RM with 1 min rest between sets of RE	• No significant change in blood lactate vs. PLA • No significant change in creatine Kinase vs. PLA • Decrease in upper and lower arm muscle soreness vs. PLA ∗Following 24, 48 and 72 h
Trexler et al. [75]	- REC males (n = 27) - Age: 22 ± 4 years	- CM: 8 g, 2-h prior to exercise	- RDB design - placebo-controlled - used to test submaximal isotonic leg extensions during 25 % of maximal voluntary contraction torque of RE for 3 min (one repetition every 4 s)	• No significant change in muscle blood flow (CM: 3.78 ± 0.26, PLA: 3.72 ± 0.26 ml min^−1^.100 ml^−1^) or oxygen consumption (CM:1.15 ± 0.11, PLA: 1.16 ± 0.11mLO_2_. min^−1^.100 g^−1^)
Trexler et al. [29]	- REC males (n = 27) - Age: 22 ± 4 years	- CM: 8 g, 2-h prior to exercise	- RDB design - placebo-controlled - used to test maximal concentric leg extensions during 5 sets × 30 reps of RE with 1 min of passive rest between sets	• Little increase in NO (CM: 15.3 ± 1.1; PLA: 13.4 ± 1.1 μmol L) • No significant change in blood flow vs. PLA • No significant change in metabolic efficiency vs. PLA • No significant change in hormonal response vs. PLA

*REC:* recreationally active*; RDB:* randomized, double-blinded; *CM*: citrulline malate; *RT:* resistance trained; *RE* resistance exercise; *RM:* repetition max; *HR:* heart rate.

### 4.2 Citrulline malate and high-intensity interval training

The effects of citrulline supplementation on high-intensity strength and power outcomes have been studied extensively in recent years [29]. In a study by Buckinx et al. [91], a total of 56 obese elderly individuals were provided with citrulline supplementation combined with HIIT to compare the effects HIIT alone versus HIIT combined with L-citrulline supplementation on functional capacity and muscle function in dynapenic-obese elderly. It was reported that citrulline supplementation combined with HIIT significantly improves upper limb muscle strength and walking speed in older adults with dynapenic obesity. These studies show small but positive effects of citrulline supplementation on high-intensity strength that are also summarized within the review by Trexler et al. [29]. Additionally, for high-level athletes whose margins of victory are small, citrulline supplementation may impart meaningful effects on strength and power athletes [63]. Working with elite athletes involves being a part of a high-performance culture where performances are measured by results, and every small improvement counts [20]. Accordingly, the relevant studies [91] highlight that athletes involved in intensive preparation for high level training or competitive events may profit from CM [88].

However, when basic ethical guidelines regarding supplements and doping are not part of the culture or communicated, athletes may be encouraged to make risky decisions in favor of performance. The lack of awareness of basic nutrition principles could also lead many athletes and non-athletes to be persuaded by advertisements from manufacturers aiming to solely benefit from the sale of supplements. Discussing physical, physiological, cultural, and ethical issues is recommended to ensure that athletes have the information they need to make informed decisions [20]. Nonetheless, despite the positive outcomes of such studies, future research should continue to fully explore the doses and effects of acute and chronic L-citrulline and CM supplementation on blood flow markers and exercise performance, along with attempting to unravel the precise mechanism behind such effects [84].

## 5 Practical implications and recommendations

This study aimed to review the mechanism associated with CM supplementation along with the implementation of different HIIT methods. That combination could help improve sports performance by increasing ATP, vasodilation, and enhancing work capacity. In the study of the effectiveness of high intensity interval training and the use of CM supplements, muscle strength and walking speed were higher in sarcopenia compared to the control group. There are few studies on the effectiveness of this supplement on sports performance.

The current review can be considered a first step in explaining the interaction between CM consumption and HIIT on exercise performance and summarizes the most relevant literature. However, the results summarized by this review article should be interpreted meticulously due to the limited details on the standard dose and timing of CM consumption in addition to the ambiguity of the mechanism of action and source of purchase to better optimize sports performance.

Consequently, it is recommended that future studies further examine the issues discussed above and expand the evidence in this regard.

## 6 Conclusions and future directions

This article reviewed the benefits, modalities, and mechanisms of action of HIIT and its interaction with CM, which functions as an ergogenic aid to optimize exercise performance during HIIT.

Ultimately, further research is required to: identify the level of plasma NO, citrulline, and arginine in different doses; to define the standard dose and timing of CM consumption in different training models and; CMs mechanism of action due to the synergistic impact of its components. This supplement should also be studied to check the effect of malate in the energy cycle. Citrulline malate is an effective supplement for increasing systemic arginine levels, but several factors can limit its effectiveness in boosting arginine production and NO synthesis. These include enzymatic saturation, competition with other pathways, high arginase activity, and impaired NOS function. Strategies like optimizing cofactor availability, reducing oxidative stress, and targeting arginase activity can help overcome these challenges and enhance the benefits of citrulline malate supplementation. These future directions would help to confirm CM's ergogenic effects. In addition, attempts to prevent the unconscious purchase and self-prescription of supplements that might result in unexpected doping and toxicity should not be underestimated.

## CRediT authorship contribution statement

**Hadi Nobari:** Writing – review & editing, Writing – original draft, Validation, Software, Data curation, Conceptualization. **Laya Samadian:** Writing – review & editing, Writing – original draft, Methodology, Investigation, Data curation, Conceptualization. **Saber Saedmocheshi:** Writing – review & editing, Writing – original draft, Data curation, Conceptualization. **Pablo Prieto-González:** Writing – review & editing, Writing – original draft, Data curation, Conceptualization. **Christopher MacDonald:** Writing – review & editing, Writing – original draft, Data curation, Conceptualization.

## Ethics approval and consent to participate

Not Applicable.

## Availability of data and materials

Not Applicable.

## Data availability statement

No data was used for the research described in the article.

## Funding

This research received no external funding.

## Declaration of competing interest

The researchers have no other conflicts of interest. And they promise not to send the article anywhere until the review is complete.

## Footnotes

1 - heart rate reserve.
